# Amino Acid Metabolism in Apicomplexan Parasites

**DOI:** 10.3390/metabo11020061

**Published:** 2021-01-20

**Authors:** Aarti Krishnan, Dominique Soldati-Favre

**Affiliations:** Department of Microbiology and Molecular Medicine, University of Geneva, CMU, Rue Michel-Servet 1, 1211 Geneva, Switzerland; Dominique.Soldati-Favre@unige.ch

**Keywords:** amino acids, apicomplexans, *Toxoplasma gondii*, *Plasmodium* spp., de novo biosynthesis, uptake, transporters, lifecycle stages

## Abstract

Obligate intracellular pathogens have coevolved with their host, leading to clever strategies to access nutrients, to combat the host’s immune response, and to establish a safe niche for intracellular replication. The host, on the other hand, has also developed ways to restrict the replication of invaders by limiting access to nutrients required for pathogen survival. In this review, we describe the recent advancements in both computational methods and high-throughput –omics techniques that have been used to study and interrogate metabolic functions in the context of intracellular parasitism. Specifically, we cover the current knowledge on the presence of amino acid biosynthesis and uptake within the Apicomplexa phylum, focusing on human-infecting pathogens: *Toxoplasma gondii* and *Plasmodium falciparum*. Given the complex multi-host lifecycle of these pathogens, we hypothesize that amino acids are made, rather than acquired, depending on the host niche. We summarize the stage specificities of enzymes revealed through transcriptomics data, the relevance of amino acids for parasite pathogenesis in vivo, and the role of their transporters. Targeting one or more of these pathways may lead to a deeper understanding of the specific contributions of biosynthesis versus acquisition of amino acids and to design better intervention strategies against the apicomplexan parasites.

## 1. Introduction

Parasites of the Apicomplexa phylum such as *Toxoplasma*, *Plasmodium*, and *Cryptosporidium* spp. cause significant morbidity and mortality in humans and in livestock [1,2,3,4]. Together, they lead to over half a million deaths each year and enormous economic burden [5,6]. The extraordinary ability of these parasites to invade, adapt, and persist in diverse host niches is multifactorial and depends on highly specialized pathogen-driven mechanisms which have evolved over a hundred million years [7,8]. Communication between a pathogen and its host therefore relies on a wide and dynamic array of molecular interactions, including exchanges of key metabolites. Although significant progress has been made in understanding host–pathogen interplay, a comprehensive and in-depth grasp of the metabolic capabilities of the various parasite developmental stages has not been achieved. Successful parasite replication is accomplished with the acquisition of essential nutrients from the host, which represents a vast repository of metabolites. However, the precise contributions of parasite de novo biosynthesis pathways versus acquisition are yet to be delineated. Recent studies further indicate that the nutritional availabilities and capabilities of the parasites vary between in vitro and in vivo conditions [9]. Therefore, to fully understand the underlying pathways, global, large-scale, and exhaustive experimental and computational approaches are becoming indispensable. 

Indeed, in the last few years, our understanding of apicomplexan metabolism has improved with the assembly and curation of parasite genomes [10]. This information has enabled the generation of genome-scale metabolic networks in particular for *T. gondii* and *Plasmodium* spp. [9,11,12,13,14], which collectively encompass all known and experimentally validated genes encoding for proteins and enzymes associated with metabolic reactions. The biochemical reactions, localized to specific subcellular compartments, ultimately lead to the production of biomass, which constitutes the objective function of the mathematical framework. The framework can then be used to perform in silico simulations and to yield insights into gene and reaction essentialities, synthetic lethality, and in silico minimal media predictions. To challenge and improve the predictive power of these models, the latest reconstructions for *T. gondii* (iTgo) and the malaria parasites (*P. falciparum* and *P. berghei* (iPfa and iPbe, respectively)) were incorporated with valuable and recently obtained global experimental data. These datasets included large-scale genome-wide information on the fitness of parasites upon gene disruption [15,16,17], transcriptomics/RNAseq profiles of genes across lifecycle stages [18,19,20], and/or qualitative and quantitative metabolomics data [21,22,23]. Conceivably, incorporation of the different layers of –omics data (genomics, transcriptomics, and metabolomics) into the metabolic networks led to improved consistency with experimental data [9,14].

More recently, an impressive dataset assigned subcellular locations to over 2500 proteins in *T. gondii* using the hyperLOPIT (hyperplexed localization of organelle proteins by isotope tagging) method [24]. For a genome-scale model such as iTgo, precise cellular localization of enzymes enables accurate predictions on the feasibility of a reaction in a given compartment. Therefore, in an iterative cycle, the integration of high-throughput –omics data into computational frameworks will improve model-guided investigations for experimental studies, data which can be fed back into the network for another round of predictions. 

Strikingly and of relevance to this review, in silico analysis on the curated and harmonized iTgo model revealed that pathways for the de novo biosynthesis of several amino acids was dispensable [9]. This led us to hypothesize that, in the fast-replicating stage of the parasite (hereafter referred to as tachyzoites), most of the amino acids were acquired from the host. Dedicated transport mechanisms for some of the amino acids have been investigated [25,26] and uptake of proteins and subsequent proteolytic degradation within a specialized vacuolar organelle (VAC) has previously been described [27]. However, transporters for several other amino acids are still unidentified, opening numerous doors for future investigation. Interestingly, the enzymes that do exist for de novo synthesis or interconversion of amino acids are transcriptionally upregulated in other developmental stages of the parasites (slow-growing bradyzoites, merozoites, gametes, or oocysts) compared to tachyzoites [18] (Table S1). 

**Figure 1:**
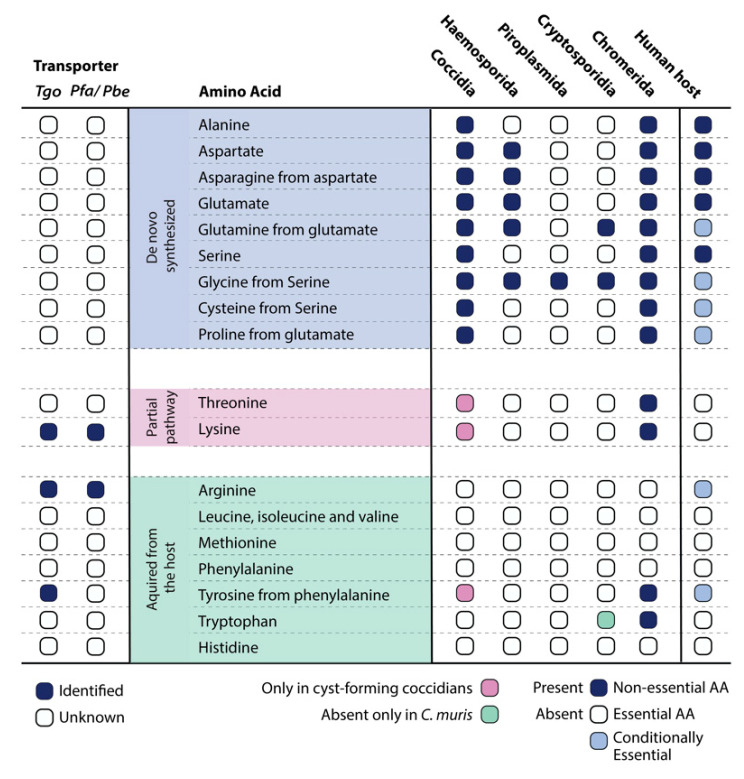
Conservation of the 20 proteogenic amino acid pathways within the Apicomplexa phylum and in the human host. On the left, identified or unidentified transporters for *T. gondii* and *Plasmodium* spp.

Genomic analysis further illustrates that, among the Apicomplexa, the cyst-forming coccidian subgroup along with *Chromeridae*, which shares a proto-apicomplexan ancestor, have the greatest number of enzymes for the synthesis of amino acids (Figure 1). As expected, in certain genera such as the *Cryptosporidia* and piroplasms, a large number of genes have been lost. This is likely reflective of their intracellular lifestyle, as *Cryptosporidia* develop in an extra-cytoplasmic compartment on the apical surface of intestinal epithelium cells and piroplasms proliferate freely within the host cytosol, enabling direct access to nutrients from the gut microbiome and host cell, respectively [28,29,30,31]. 

In the next sections, derived from our analysis on the iTgo metabolic network for the tachyzoite stage, we discuss the status of all 20 proteogenic amino acids and a few non-proteogenic amino acids within the Apicomplexa phylum. We place special emphasis on the cyst-forming coccidian *T. gondii* as well as on the vector-born *Plasmodium* spp. and hope to unravel the presence or absence of biosynthetic capabilities, the transport mechanisms, and their relevance during the distinct lifecycle stages. In several cases, we show how cutting-edge technologies such as ^13^C labeling and metabolomics approaches help delineate the contributions of de novo synthesis versus uptake of this important class of metabolites. 

## 2. Amino Acids That Can Be Synthesized De Novo

### 2.1. Alanine

Alanine is a nonessential amino acid synthesized by humans. The two-step synthesis of alanine from pyruvate, however, is unique only to the coccidian subgroup of the Apicomplexa (Figure 1). Alanine dehydrogenase and alanine transaminase are the enzymes responsible for this reaction. A ^13^C glucose-labeling analysis on *T. gondii* tachyzoites showed the de novo synthesis of alanine [32]. Alanine racemase, on the other hand, is a pyridoxal-5-phosphate (PLP)-dependent enzyme that catalyzes the isomerization of L-alanine to D-alanine and is present in both the *Plasmodium* spp. and *T. gondii* genomes, although the L-isomer of alanine is the one incorporated into proteins, with D-alanine being a rare form. D-alanine occurs in polypeptides of some bacterial cell walls [33], but its role in apicomplexan parasites remains unknown. 

Based on the CRISPR-Cas9 screen that assigns fitness scores (FS) for every gene, ranging from +3 (indicating dispensability) to −7 (indicating severe fitness loss or a fitness-conferring phenotype) [17], the enzymes for alanine biosynthesis and isoform interconversion show dispensability. This indicates the uptake of L-alanine (apart from its synthesis) from the host. No transporter has been identified to date; however, the incubation of tachyzoites with ^13^C-labelled amino acids showed the uptake of labeled alanine into the parasite [25]. Therefore, given the two routes to obtain alanine in coccidian species, targeting the synthesis enzymes and transporter, when identified, could result in synthetic lethality. 

Contrastingly, during the erythrocytic stages of *P. falciparum,* alanine was shown to be taken up but competed with isoleucine and methionine, suggesting that the unidentified transport mechanism was unspecific to neutral amino acids [34]. The transport mechanism, either via a transporter or hemoglobin degradation, must however be essential as Haemosporidians lack biosynthesis capabilities, yet this hypothesis remains to be tested.

### 2.2. Asparagine and Aspartate

**Figure 2:**
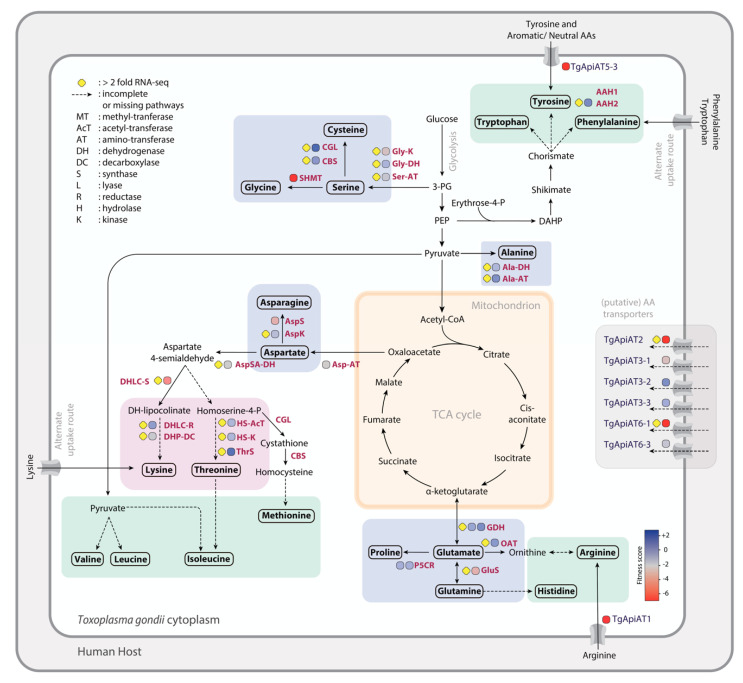
Amino acid biosynthesis and acquisition pathways in *T. gondii* parasites: the enzymes are indicated in dark purple, with their fitness scores (FS) ranging from blue (dispensable) to red (fitness-conferring). Yellow diamonds indicate genes with >2 log2FC (fold-change) in a different developmental stage compared to tachyzoites. Fold-change values can be found in Table S1. Abbreviations: AAH, aromatic amino acid hydrolase; ApiAT, apicomplexan amino acid transporter; CGL, cystathione gamma lyase; CBS, cystathionine beta-synthase; SHMT, serine hydroxymethyltransferase; 3-PG, 3- phosphoglycerate; PEP, phosphoenolpyruvate; DAHP, dihydroxyacetone phosphate; TCA, tricarboxylic acid; AspSA, aspartate semialdehyde; DHLC, dihydrodipicolinate; DHP, diaminopimelate; HS, homoserine; GDH, glutamate dehydrogenase; OAT, ornithine aminotransferase; P5C, pyrroline-5-carboxylate.

In most organisms, asparagine and aspartate are nonessential amino acids and play important roles in the context of glycoprotein formation, as asparagine residues are typically key sites for N-linked glycosylation. In all species of coccidians and haemosporidians, aspartate (also a precursor for asparagine) can be synthesized from oxaloacetate, using a transaminase enzyme. Transaminase transfers the amino group from glutamate to oxaloacetate, producing α-ketoglutarate and aspartate (Figure 2). A metabolic labeling analysis with ^13^C glucose showed significant de novo synthesis of aspartate [32]. From aspartate, the asparagine synthetase can then produce asparagine. Aspartate aminotransferase in *P. falciparum* (PfAspAT) was demonstrated to have in vitro and in vivo activity [35]. Asparagine synthetase (PbAspS) was also shown to be active and important for both the asexual and sexual lifecycle stages of *P. berghei* in vivo, with drastic reduction in sporozoite numbers within the mosquito [36]. 

Transporters for aspartate and asparagine have not been identified, and only an uptake of very low amounts of ^13^C labelled amino acids was observed in *T. gondii*, suggesting a predominant and active biosynthesis in the tachyzoite stage [25]. However, the FS of these enzymes show dispensability (Figure 2), indicating that uptake may occur under rich-media conditions when the biosynthesis pathway is perturbed. Culturing the parasites in minimal media or media lacking aspartate might make the biosynthesis enzymes essential. 

Aspartate is also required for the production of adenylosuccinate and *N*-carbamoyl-aspartate (precursors of purine and pyrimidine biosynthesis, respectively), and the enzymes implicated in this catalysis are adenylosuccinate synthase and aspartate carbamoyltransferase. The crystal structure of aspartate carbamoyltransferase (also termed aspartate transcarbamoylase (PfATC)) has been resolved and was shown to be catalytically active [37]. The enzyme was also validated as a potential drug target against *P. falciparum* [38,39], suggesting essential downstream functions of this amino acid. HyperLOPIT subcellular localization data for *T. gondii* indicates that all the enzymes are present in the cytosol and points to synthesis and utilization within this compartment (Table S1) [24]. 

Among the other apicomplexans, *Cryptosporidia* and piroplasms do not possess any enzymes to make asparagine or aspartate and must rely solely on uptake from their respective host niches (Figure 1). When identified, the transporters could represent essential targets for therapeutic intervention. 

### 2.3. Glutamate and Glutamine

Glutamate and glutamine are nonessential amino acids for both *T. gondii* and *P. falciparum*, as is the case for humans. Most organisms possess a NAD^+^ (nicotinamide adenine dinucleotide) and NADP^+^ (nicotinamide adenine dinucleotide phosphate)-specific glutamate dehydrogenase that catalyzes the reversible conversion of glutamate to 2-oxoglutarate. In *T. gondii,* numerous studies with ^13^C labeled glucose indicated the de novo synthesis of glutamine from glucose via α-ketoglutarate [32,40,41,42] (Figure 2). Glutamine and glutamate can also be interconverted via the action of glutamate ammonia-ligase/glutamine synthetase and can enter the mitochondrion to fuel the lower TCA cycle. In freshly egressed extracellular tachyzoites, it was shown that the parasites co-utilize glucose and glutamine as carbon sources but can also survive on glutamine alone when glucose uptake is compromised [32,41,43]. GABA (γ-aminobutyric acid) derived from glutamine also acts as a short-term energy reserve to sustain ATP levels for parasite motility and invasion [32]. Further, quantitative ^13^C-NMR analysis of the supernatant of extracellular tachyzoites (fed with ^13^C-labeled glucose) revealed that glutamate, alanine, and aspartate were secreted as metabolic end-products at this non-dividing step of the lytic cycle rather than incorporated into the biomass [32]. This suggests that excess amounts of amino acids in non-dividing cells can lead to toxic effects and must be eliminated to maintain metabolic homeostasis [32].

Interestingly and in contrast to what was previously thought for the erythrocytic stages of *P. falciparum*, several metabolites were derived from both ^13^C labeled glucose and glutamine [44]. The TCA cycle intermediates were predominantly labeled from glutamine; however, in the gametocyte stages, labeling of the TCA cycle from glucose was increased dramatically due to the higher glucose uptake rate. This observation indicates a high-energy requirement of the sexual stage and that the TCA cycle is functional, utilizing both glucose and glutamine [44]. In *Plasmodium* spp., glutamate can also be produced via the degradation of arginine [45], a pathway that is missing in *T. gondii,* as no arginase could be found encoded in its genome. 

Given the redundancy of the many pathways to make glutamate and potential uptake of the amino acid from the host, it is expected that the above enzymes are dispensable or are associated with a mild fitness defect. Concordantly, this is reflected in the FS in *T. gondii* (Figure 2) and in the PlasmoGEM screen on the asexual blood stages of the *Plasmodium* spp. [16]. The transporters for glutamine and glutamate however remain elusive, and it would be of interest to test the synthetic lethality of the transporter(s) and/or key enzymes in the biosynthesis pathway in both apicomplexans. 

Glutamate is a versatile amino acid, acting as a precursor for several downstream metabolites. L-proline is synthesized from glutamine via L-glutamate-5-semialdehyde. The genes encoding the two enzymes of the pathway glutamate 5-kinase and glutamyl phosphate reductase are present in all coccidians but absent in *Plasmodium* spp. The precursors for aminosugars and pyrimidine biosynthesis, Glucosamine-6P and Carbamoyl-5P, respectively, are also produced from glutamine via the action of hexose-phosphate aminotransferase and carbamoyl-phosphate synthase. These capabilities are present in *T. gondii* but absent in *Plasmodium* spp. The enzyme to convert uridine-5-triphosphate into cytidine triphosphate (CTP) also utilizes glutamine, and recombinant CTP synthase in *T. gondii* has been purified and experimentally shown to be essential for parasite survival [46]. 

Lastly, *T. gondii* also encodes the pathway for nitrogen metabolism that involves the utilization of nitrate and nitrite from the host as a source of ammonia. Ammonia is required for the de novo synthesis of glutamine and glutamate, yet transporters importing nitrite and nitrate have not been formally characterized in *T. gondii* to date. The genome however encodes three formate-nitrate transporters (FNTs) that were shown to play a major role in the efflux of L-lactate in both *T. gondii* and *P. falciparum* [46,47,48]. Whether synthesized or taken up from the host, glutamate and glutamine remain essential amino acids for diverse cellular processes and are required for the production of key vitamins (pantothenate, folate, and pyridoxal-phosphate) and cofactors (CoA, heme, glutathione, and s-adenosylmethionine), highlighting their importance for parasite survival. 

### 2.4. Glycine, Serine, and Cysteine

*T. gondii* possesses the ability to synthesize glycine, serine and cysteine. Serine is made with glycerate-3-phosphate as the substrate (Figure 2) and requires the action of the following enzymes: glycerate kinase, phosphoglycerate dehydrogenase, phosphoserine transaminase, and phosphoserine phosphatase. However, the FS of these enzymes indicate dispensability, and predictably, ^13^C glucose labelling did not lead to serine being labelled in tachyzoites (Joachim Kloehn, personal communication). This observation is further supported by efficient uptake of stable-isotope-labelled serine [25]. Hence, whether the synthesis pathway is active in another stage of the lifecycle, such as the sexual or oocyst stages, remains to be investigated. In contrast with *T. gondii*, the *Plasmodium* spp. genome lacks all the enzymes for the synthesis of serine and is thus auxotrophic for the amino acid. 

Once taken up, serine can be interconverted into cysteine in an unusual pathway present only in coccidian species via cystathionine beta-synthase and cystathionine gamma-lyase. Homocysteine (along with serine) is a precursor and can be synthesized in a multi-step process. First, methionine receives an adenosine group from ATP, catalyzed by S-adenosyl-methionine synthetase, giving rise to S-adenosyl methionine (SAM). SAM then transfers the methyl group to an acceptor molecule, S-adenosyl-homocysteine (SAH), and the adenosine is hydrolyzed via adenosylhomocysteinase to yield l-homocysteine. l-homocysteine has two primary fates: conversion via tetrahydrofolate (THF) back into l-methionine (a capability lacking in *T. gondii*) or conversion to l-cysteine. 

l-cysteine along with l-valine can then fuel the pathway for biosynthesis of an essential cofactor, CoA, and its precursor, pantothenate, respectively [49]. Cysteine along with glutamate and glycine are also used to make the redox intermediate, glutathione (GSH). GSH can be de novo synthesized by *T. gondii,* as the parasite possesses the two enzymes gamma-glutamylcysteine synthetase and glutathione synthetase. Therefore, apart from their proteogenic use, the participation of these amino acids in other metabolic pathways account for their importance in overall cellular fitness and survival.

Interestingly, in all apicomplexans, glycine can be interconverted from serine via serine hydroxymethyltransferase (SHMT) (Figure 1). The activity of SHMT to produce glycine was experientially demonstrated in *P. falciparum* [50,51], and the FS of the enzyme for tachyzoites indicates its essentiality for parasite survival. However, in *T. gondii*, ^13^C glucose labeling did not demonstrate ^13^C incorporation into glycine [52]. Since SHMT also participates in one-carbon (1C) metabolism, comprising a series of interconnected pathways that include the methionine cycle and the folate cycle, its essential function may be attributed to it. The one-carbon pathway is central to cellular function, providing 1C units (methyl groups) for the synthesis of DNA, polyamines, amino acids, and phospholipids. In *T. gondii*, as in most cells, serine is also incorporated into phospholipids as phosphatidyl-serine (PtSer), an integral component of the membrane. In extracellular tachyzoites, radiolabeled serine led to labeled PtSer, providing further evidence for its efficient incorporation [41].

Finally, glycine along with succinyl-CoA can be metabolized into aminolevulinic acid (5-Ala), a precursor for the production of heme, which is a key cofactor for several reactions. Recent studies have unraveled the importance of heme biosynthesis in both *T. gondii* [9,53] and its dispensability in the erythrocytic stage of *P. falciparum* [54,55,56]. Putative transporters for serine, glycine, or cysteine have not been identified to date, although it has been suggested that these amino acids share the same uptake route as leucine or isoleucine in the asexual blood stages of *P. falciparum* [34]. Identification of the transporters and the relevance of the biosynthesis routes, possibly in a different lifecycle stage of the parasite, are therefore crucial. The enzymes to make serine and cysteine, but not glycine, are indeed upregulated in the merozoite, gametes, and oocyst stages of *T. gondii* (Table S1) [18], reflecting the importance of the pathway under nutrient-limited conditions. 

### 2.5. Proline

Proline can be de novo synthesized from glutamate in both humans and *T. gondii*. However, in *Plasmodium* spp., an alternate and unusual route for proline biosynthesis occurs via arginine degradation. Arginine is first to be broken down into ornithine via the arginase enzyme. Ornithine, via the actions of the ornithine aminotransferase (OAT) and pyrroline-5-carboxylate reductase enzymes, is then converted into proline. The *P. falciparum*, PfOAT was expressed in *E. coli* and was investigated for its activity [57,58]. PfOAT was shown to be bidirectional, functioning predominantly in the ornithine to proline direction [57]. However, as for all amino acids, given their abundance after hemoglobin digestion in the intraerythrocytic stages, the essentiality of this interconversion remains to be tested. Most likely, both coccidians and haemosporidians can take up proline from their hosts and can also utilize their biosynthesis capabilities (from glutamate and/or arginine) in a different developmental stage, where proline becomes limiting. Consistent with this, the FS of the proline biosynthesis enzymes indicate dispensability in the tachyzoite stage and are highly expressed in the merozoite or oocyst stages of *T. gondii* [18]. The transporter for proline has however not yet been identified but testing the synthetic lethality of the uptake (either by depleting the amino acid in the media or by genetically ablating the transporter along with the enzymes of the biosynthesis pathway) in one or more lifecycle stages would be of interest.

## 3. Essential Amino Acids with an Incomplete De Novo Synthesis Pathway

### 3.1. Threonine

In humans, threonine is one of the nine essential amino acids which must be obtained from the diet. *T. gondii* along with other cyst-forming coccidians possess the genes for four out of the five enzymes involved in threonine synthesis from aspartate. The known and annotated enzymes are aspartate kinase (AK), aspartate semi-aldehyde dehydrogenase (ASDH), homoserine kinase (HK), and threonine synthase (TS), with AK and ASDH being common to the threonine and lysine synthesis pathways. The missing enzyme of the threonine synthesis pathway is homoserine dehydrogenase. In a recent unpublished study, *T. gondii* ASDH and HK were shown to complement the corresponding yeast mutants, auxotrophic for threonine, highlighting their enzymatic activity. The overexpression of TgTS also led to parasites surviving the depletion of threonine in the media [59]. However, TgTS could be successfully knocked-out in the presence of threonine, indicating an efficient uptake of this amino acid in tachyzoites [59].

Thus, the existence of a fully functional pathway for threonine biosynthesis is plausible, although concrete experimental evidence is lacking and requires further investigation. Future studies must identify the missing enzyme or an alternate route to fill the gap. The FS of the known genes indicates dispensability, and therefore, tachyzoites likely take up threonine from their host. Whether the (partial) pathway is fully functional at a different developmental stage, when threonine is limited, remains to be tested. A threonine transporter has also not yet been identified but must be essential in *Plasmodium* spp., as these species rely solely on acquisition, as in humans.

### 3.2. Lysine

The diaminopimelate pathway is one of the four different routes to produce L-lysine from aspartate in a cell. Of these, three variants belong to different groups of prokaryotes and the fourth variant was recently identified in *Arabidopsis thaliana* [60]. The first four enzymes, aspartate kinase, aspartate semialdehyde dehydrogenase, dihydrodipicolinate synthase, and dihydrodipicolinate reductase, and the last enzyme, diaminopimelate decarboxylase, are common to all variants of the pathway and are present in the genome of *T. gondii*. However, the pathway comprises two other intermediate enzymes: tetrahydrodipicolinate *N*-acetyltransferase and diaminopimelate epimerase, both of which seem to be missing in *T. gondii*. A homology-based search has not led to their identification. The presence of a partial pathway is unusual, as neither of the intermediates are known to act as important precursors for other cellular processes. Errors in gene models or the presence of an alternate but highly divergent route cannot be excluded. Of relevance, labelling with the ^13^C-amino acid mix showed significant uptake and its transporter has also been well characterized in both *T. gondii* and *P. berghei.* TgApiAT6-1 was shown to unidirectionally transport lysine and, to a lesser extent, arginine and was essential for the tachyzoite stage [26,61]. However, its counterpart in the rodent malaria parasite, PbNPT1, was dispensable for the asexual blood stages, given that lysine can be obtained from hemoglobin degradation [26]. Whether the cationic amino acid transporter is required for progression during the sexual mosquito or liver stages of these parasites remains to be determined. 

## 4. Essential Amino Acids That Must Be Taken Up

### 4.1. Arginine

In humans, arginine is classified as a semi-essential or conditionally essential amino acid as its biosynthesis depends on the developmental stage and health status of an individual [62]. Arginine can be synthesized from citrulline via the sequential action of the enzymes argininosuccinate synthetase and argininosuccinate lyase. These enzymes are however absent in all apicomplexan genomes, rendering them auxotrophic for arginine. In *T. gondii*, it has been demonstrated that arginine is indeed taken up via TgApiAT1 (formerly TgNPT1) and was shown to be essential both in vitro and in vivo [25,26,61]. Parasite growth could be rescued if sufficient arginine is added to the media, indicating the presence of another low affinity transporter, which was later identified as TgApiAT6-1 [61].

Arginine, once inside the cell, can be metabolized into ornithine and carbamoyl phosphate via citrulline in reverse reactions. The enzymes involved are arginine deiminase and ornithine carbamoyl transferase, present in *T. gondii*. Importantly, it has been proposed that arginine catabolism could divert host arginine away from nitric oxide (NO) synthesis, thereby protecting the parasite from the host’s cytotoxic defense mechanism [63]. In a parallel scenario, studies have indicated that the virulent *T. gondii* strain RH (Type I) can also initiate arginine starvation by secreting a specialized parasite effector protein, ROP16. This secreted parasite kinase phosphorylates and activates STAT6, resulting in the expression of host arginase-1 [64]. Arginase-1 is a host enzyme that degrades arginine, limiting the availability of NO, and leads to a reduction in parasite growth. Given that *T. gondii* is an arginine autotroph, starvation of this amino acid leads to the conversion of tachyzoites to slow growing, bradyzoites [65], thus displaying a clever trade-off strategy by reducing replication and virulence, ultimately ensuring parasite transmission.

A similar mechanism contributing to the decrease in NO production and deformability of the infected erythrocytes was also observed in *P. falciparum.* The arginine pool of the host is sequestered via a cationic amino acid transporter (PfNPT1) and metabolized by the parasite [66]. In the hepatic stage of *Plasmodium* spp., where parasites cannot rely on hemoglobin degradation to obtain their amino acids, normal development of *P. berghei* was largely dependent on the arginine transporter PbNPT1 [26]. Separately, in a more recent in vivo study, dietary supplementation of mice with a combination of arginine (R) and two additional amino acids, lysine (K) and valine (V), termed RKV, significantly decreased *Plasmodium* late liver-stage infection [67]. It was shown that exogenously supplied RKV stimulates the host’s overall innate immune response, suggesting that amino acids, apart from their metabolic roles for cell survival, can also act as activators of downstream inflammatory signaling pathways and can be exploited for intervention against a specific lifecycle stage [67].

### 4.2. Leucine, Isoleucine, and Valine

The pathway for de novo synthesis of the branched chain amino acids leucine, isoleucine, and valine from pyruvate is present in both animals and plants. Strikingly, no member of the Apicomplexa is able to synthesize them (Figure 1), but nevertheless, the genome of *T. gondii* encodes the enzymes to degrade them into (R)-methyl-malonyl-CoA and propionyl-CoA. This pathway resides within the mitochondrion, as seen by the localization of the first two enzymes (branched-chain amino acid aminotransferase (BCAT) and branched-chain α-ketoacid dehydrogenase (BCKDH)) [42]. Propionyl-CoA is a toxic biproduct that *T. gondii* can detoxify into pyruvate via a functional 2-methycitrate cycle [68]. The enzymes coding for the 2-MCC also localize to the mitochondrion and are present in all coccidians but are absent in the other subgroups of the phylum that congruently do not produce propionyl-CoA. 

Transporters for these amino acids have not been characterized in *T. gondii* to date. However, the uptake of isoleucine and the concomitant secretion of leucine has been studied in *P. falciparum* [69]. Isoleucine is the only amino acid that cannot be obtained from hemoglobin degradation in the erythrocytic stage of *Plasmodium* spp. [34,70], and it has been shown that *P. falciparum* responds to the starvation of isoleucine by entering into hibernation [71]. Interestingly, recent experiments have revealed that isoleucine-deprived parasites never exit the cell cycle (no cytokinesis) but instead continuously grow at a markedly reduced pace [72]. However, if isoleucine is withdrawn after the onset on the S-phase (after DNA replication), the parasites continue progression at a normal pace. This behavior marks a previously undescribed nutrient-sensitive stage in the *P. falciparum* cell cycle that has important implications on parasite growth and adaptation in response to environmental changes [72]. Identification of the transporters and cellular effects upon amino acid deprivation are therefore crucial to understand and limit parasite pathogenesis. 

### 4.3. Methionine

Methionine is an essential amino acid for humans, and no biosynthesis enzymes exist in any apicomplexan genome, hence requiring it to be salvaged from the external environment. Importantly, after its acquisition, the metabolism of methionine is essential for providing two important substrates for cellular functioning: homocysteine and S-adenosyl-methionine (SAM). A putative amino acid transporter (TgApiAT6-2) was shown to transport a SAM-analog, Sinefugin [25,73], and was hypothesized to transport methionine as well, although the transporter was dispensable for parasite survival. Given that *T. gondii* and other apicomplexans are methionine auxotrophs, the transporter is expected to be highly fitness-conferring, and future studies are needed to elucidate the identity of this protein or alternate transport mechanisms. 

### 4.4. Phenylalanine, Tryptophan, and Tyrosine

The three aromatic amino acids are essential in most organisms and must be salvaged from the host. As in humans, the imported phenylalanine and tyrosine can produce L-DOPA, a precursor for dopamine (a neurotransmitter) via the two nearly identical isoforms of aromatic amino acid hydroxylase (AAH1 and AAH2). In previous studies, it was hypothesized that *T. gondii* can alter the dopamine levels in mouse brains and manipulate the behavioral response of the intermediate host. Recent work has, however, revisited the roles of TgAAH1 and TgAAH2 in this process and indicated that parasites lacking these enzymes had no significant growth defect in vitro and in vivo, neither in tachyzoites nor in bradyzoites [74,75]. Since L-DOPA is also a component of the oocyst cyst wall, the activities of AAH1 and AAH2 are speculated to be important at this stage [76]. In *T. gondii,* tyrosine was shown to be transported via a member of the ApiAT family TgApiAT5-3 [25]. Parasites lacking this transporter displayed a fitness-conferring phenotype based on its FS but could be rescued if excess tyrosine was added to the media. Interestingly, rescue could also be observed when intermediate concentrations of phenylalanine and tryptophan were supplemented [25], thus reflecting the presence of an alternate amino acid transporter, primarily transporting phenylalanine and tryptophan, with lower affinity to tyrosine. 

In a different study, phenylalanine was confirmed to be taken up via stable isotope labelling of amino acids in cell culture (SILAC) [77]. The method was combined with Raman micro-spectroscopy to selectively monitor the incorporation of deuterium-labelled phenylalanine [l-Phe(D8)] into proteins. Interestingly, only in intracellular tachyzoites, [l-Phe(D8)] was completely replaced by I-Phe 7–9 h post infection while extracellular parasites were unable to incorporate the labeled amino acid even after 24 h [77]. This suggests that *T. gondii* tachyzoites exclusively take up phenylalanine from the host while intracellular, although the identity of the transporter has not been elucidated to date. 

In regard to tryptophan, *T. gondii* has been shown to induce the uptake of this amino acid in infected cells by upregulating the known human transporter, LAT-1 [78]. However, in infected cells, IFN-γ-inducible indole-2,3-dioxygenase 1 (IDO1) is upregulated and mediates the production of regulatory T-cells (Treg) via the aryl hydrocarbon 4 receptor (AhR) [78,79,80]. AhR is ligand-dependent and binds to several metabolites of the tryptophan catabolic pathway to suppress the immune response. Intestinal bacteria have been shown to degrade tryptophan into the ligands of the AhR. Curiously, some studies have shown that eliminating gram-negative bacteria in the gut microbiome of mice with antibiotics led to excess tryptophan in the environment and reduced expression of AhR, protecting the mice against an acute infection with *T. gondii* [81].

In the case of *Plasmodium* spp., studies have suggested that the parasites induce tryptophan degradation in dendritic cells to suppress the immune system and to evade immune detection [82,83]. Concordantly, within immune cells, T-cell proliferation was shown to be decreased when the pathway for tryptophan degradation was active [84], suggesting distinct host–pathogen mechanisms that connect tryptophan metabolism to immune response. 

Within the apicomplexans, a tryptophan transporter has not been identified, although, surprisingly, an enzyme for tryptophan biosynthesis was identified in the genome of a few species of the *Cryptosporium* subphylum. Tryptophan can be made from serine and indole rings via tryptophan synthase (TrpS), and both *C. parvum* and *C. hominis* possess the corresponding gene. Contrastingly, *C. muris* is one of the subspecies that lacks TrpS (Figure 1) and it is hypothesized that this difference could be attributed to the diverse niche of these organisms. *C. parvum* and *C. hominis* inhabit epithelial cells of the small intestine, while *C. muris* occupies the gastric glands of the stomach. It is therefore plausible that the amount of indole is scarce in the stomach, leading to *C. muris* relying directly on the uptake of the amino acid. Testing the essentiality of the enzyme and identifying the transporter in the other apicomplexans would be crucial to understanding both the metabolic and immune-mediated relevance of this aromatic amino acid. 

### 4.5. Histidine

Histidine is an essential amino acid for most cells. No enzymes for its de novo biosynthesis could be identified within the Apicomplexa phylum, and no transport mechanism has been identified to date. However, in oocysts expressing TgApiAT5-3 (tyrosine transporter in *T. gondii*), the uptake and secretion of ^14^C-labelled histidine was shown [25]. This indicates that TgApiAT5-3 acts as an exchanger, both enabling the uptake of tyrosine into the parasite and maintaining intracellular pools of aromatic and large neutral amino acids such as histidine [25]. Elucidating the transport mechanisms that must be essential for both *T. gondii* and *Plasmodium* spp. in all their lifecycle stages would open doors for new drug targeting candidates against these pathogens. 

## 5. Non-Proteogenic Amino Acids

Non-proteinogenic amino acids are those that are not naturally encoded by the genetic code of any organism. Over 140 non-proteinogenic amino acids are known and play a crucial role in cellular functions, acting as intermediates in biosynthesis pathways, neurotransmitters, and toxins and even as components of bacterial cell walls [85]. Interestingly, several of them can be synthesized by *T. gondii* and *Plasmodium* spp. Notable examples are 4-aminobenzoic acid (PABA) [86,87], γ-Aminobutyric acid (GABA) [32], aminolevulinic acid (5-ALA) [9,53], homocysteine, and homoserine [13]. Others, such as citrulline, diaminopimelic acid, ornithine, and β-alanine, however, are predicted not to be synthesized by *T. gondii* and must be taken up from the host. Future studies would be needed to identify the transport mechanisms and essentiality of the synthesis of non-proteogenic amino acids for parasite survival. 

## 6. Discussion

Amino acids are vital to survival, not only to make up proteins but also as key molecules for the proper functioning of metabolic pathways. Recent technologies have enabled us to understand the importance of the biosynthesis versus acquisition routes of amino acids in human-infecting pathogens. A combination of experimental and computational approaches, such as genome-wide metabolic models, curated and harmonized with CRISPR-Cas9 screening, transcriptomics, and metabolomics data, have been instrumental in deepening our understanding of the underlying mechanisms. Using our genome-scale metabolic model for the fast-replicating stage of *T. gondii*, iTgo, we analyzed the pathways for the biosynthesis of amino acids. A comparison of our model predictions with experimental CRISPR-Cas9 screening data revealed that enzymes to produce amino acids in the tachyzoite stage were dispensable, leading us to hypothesize the utilization of these enzymes in a different developmental stage [9]. This observation was indeed supported by the increased transcript levels in the bradyzoite, merozoite, gamete, and oocysts of *T. gondii* [18].

Thorough investigations of each of the 20 amino acids disclosed the existence of two (partial) biosynthesis pathways for threonine and lysine within the parasite, but they were found missing in the human host. This highlights potential opportunities for drug discovery and development, specifically targeting the parasite’s metabolism. Moreover, by comparing the genomes of species within the Apicomplexa phylum, the non-cyst forming branch (*Eimeria and Cyclospora* spp.) lacks the enzymes for threonine and lysine biosynthesis (Figure 1). It likely indicates the need for de novo synthesis of these amino acids during encystation and calls for future work to identify the missing enzymes and their importance during latency or a different developmental stage. Fascinatingly and unique to a few apicomplexans, *Cardiosporidium cionae* appears to have maintained a bacterial endosymbiont that contains the pathway for de novo lysine biosynthesis [88]. It provides only two key metabolites: lysine and lipoic acid, a mechanism that cleverly reduces host dependency and potential virulence, making the extracellular life of *C. cionae* possible [88].

Although capable of synthesizing quite a few amino acids (alanine, aspartate, asparagine, glutamine, glutamate, glycine, cysteine, and proline), apicomplexan parasites, at least in their fast-replicating stages, primarily rely on uptake from the host. Especially during the intraerythrocytic stage, the amino acids requirement of the malaria parasites is largely met via host-derived hemoglobin degradation within a digestive vacuole (DV) [71,89]. Several aminopeptidases are known to be harbored within the DV, where breakdown proteins generate short peptides and amino acids, which can be transported out of the DV [90,91]. Given such a major reliance on the host for amino acids, identifying the transporter proteins and other amino acid acquisition mechanisms will prove to be of significant interest for drug targeting. According to Parker et al., the transmembrane transporters identified in *T. gondii* share the classical domains but are largely unspecific (transporting more than one amino acid) [25]. The study included 16 transporter-like proteins (Table S2); however, experimental data suggested that only a few displayed a fitness-conferring phenotype [25]. The dispensable transporters therefore represent potential candidates for which the parasite has biosynthesis capabilities or redundant transport mechanisms. Performing high-throughput functional screens to identify synthetic lethality between the known enzymes and/or transporters could hence yield valuable information.

Most apicomplexans possess two endosymbionts: a unique relic of a plastid organelle called the apicoplast, that resulted from an ancient secondary endosymbiosis event and a single tubular mitochondrion. Both of these organelles harbor several essential metabolic pathways and utilize amino acids for their proper functioning. In order to fulfill the translational requirements of these organelles, in addition to the cytosolic machinery, the parasite must maintain a supply of charged tRNAs. Aminoacyl-tRNA synthetases (aaRSs) play an essential role in this process by charging the tRNAs with their cognate amino acids. Interestingly, in *T. gondii*, the apicoplast translation seems to be adequate with its own set of tRNAs and imported aaRSs, while the mitochondrion relies exclusively on the cytosolic components [92]. However, given the essentiality of the aaRSs for ribosomal protein synthesis, several of them have been suggested and characterised as promising antiparasitic targets against coccidians, haemosporidians and cryptosporidium [93,94,95]. Inhibiting the transport of the amino acids and charged tRNAs to the endosymbiotic compartments could further present novel drug targets for intervention.

As highlighted, it is well known that amino acids are essential for protein production, but more recently, some light has been shed on the interactions between amino acid metabolism and modulation of the host’s immune response. In vivo studies in mice showed that supplementation of a cocktail of amino acids (arginine, lysine, and valine (RKV)) during the liver stage of *Plasmodium* parasites abolished proliferation, as the amino acids played a crucial role in boosting the innate immune response. RKV activated MyD88, a crucial adaptor molecule involved in Toll-like receptor (TLR)-mediated signal transduction, essential for the function of myeloid cells and subsequent parasite elimination [67].

In parallel studies on cerebral malaria, the most severe symptom of malaria, where parasites destroy the blood–brain barrier, arginine supplementation was shown to increase cerebral blood flow, thereby partially reversing the several damages caused by cerebral ischemia [96]. These observations warrant further exploration of metabolite-based strategies against the apicomplexans and focus on the interface between amino acid metabolism and eradication of parasite burden. 

Lastly, little knowledge has been gained on the status of amino acids in the chronic, slow-growing and dormant stages of both *T. gondii* and *Plasmodium* hypnozoites. Therefore, future work must focus on constructing in silico stage-specific metabolic networks and on generating experimental data to shed light on these enigmatic stages. Targeting either the biosynthesis capabilities or acquisition pathways or both would enable better intervention strategies that could put an end not only to parasite proliferation but also to persistence and transmission of these disease-causing pathogens.

## Data Availability

The data presented in this study are available in the Supplementary files (Tables S1 and S2).

## Supplementary Materials

The following are available online at https://www.mdpi.com/2218-1989/11/2/61/s1. Table S1. Information of the enzymes participating in amino acid biosynthesis and metabolism (FS from the CRISPR-screen, hyperLOPIT data, and RNAseq transcript fold-changes compared to *T. gondii* tachyzoites and conservation within the apicomplexan phylum). Table S2. Information on the known amino acid transporters in *T. gondii* (TgApiAT family of transporters).
